# Availability of Cognitive Resources in Early Life Predicts Transitions Between Cognitive States in Middle and Older Adults From Europe

**DOI:** 10.1093/geroni/igad124

**Published:** 2023-10-26

**Authors:** Nathan A Lewis, Tomiko Yoneda, René J F Melis, Daniel K Mroczek, Scott M Hofer, Graciela Muniz-Terrera

**Affiliations:** Institute on Aging and Lifelong Health, University of Victoria, Victoria, British Columbia, Canada; Department of Psychology, University of British Columbia, Vancouver, British Columbia, Canada; Department of Medical Social Sciences, Feinberg School of Medicine, Northwestern University, Chicago, Illinois, USA; Department of Psychology, University of California, Davis, California, USA; Department of Geriatric Medicine, Radboud Institute for Health Sciences, Radboud University Medical Center, Nijmegen, The Netherlands; Department of Medical Social Sciences, Feinberg School of Medicine, Northwestern University, Chicago, Illinois, USA; Pacific Health Research and Education Institute, Honolulu, Hawaii, USA; Department of Neurology, School of Medicine, Oregon Health & Science University, Portland, Oregon, USA; Ohio University Heritage College of Osteopathic Medicine, Ohio University, Athens, Ohio, USA; Edinburgh Dementia Prevention, University of Edinburgh, Edinburgh, UK

**Keywords:** Cognitive reserve, Cognitive resilience, Cognitive resources

## Abstract

**Background and Objectives:**

The existing literature highlights the importance of reading books in middle-to-older adulthood for cognitive functioning; very few studies, however, have examined the importance of childhood cognitive resources for cognitive outcomes later in life.

**Research Design and Methods:**

Using data from 11 countries included in the Survey of Health, Ageing, and Retirement in Europe (SHARE) data set (*N* = 32,783), multistate survival models (MSMs) were fit to examine the importance of access to reading material in childhood on transitions through cognitive status categories (no cognitive impairment and impaired cognitive functioning) and death. Additionally, using the transition probabilities estimated by the MSMs, we estimated the remaining years of life without cognitive impairment and total longevity. All models were fit individually in each country, as well as within the pooled SHARE sample.

**Results:**

Adjusting for age, sex, education, and childhood socioeconomic status, the overall pooled estimate indicated that access to more books at age 10 was associated with a decreased risk of developing cognitive impairment (adjusted hazard ratio = 0.79, confidence interval: 0.76–0.82). Access to childhood books was not associated with risk of transitioning from normal cognitive functioning to death, or from cognitive impairment to death. *Total* longevity was similar between participants reporting high (+1 standard deviation [*SD*]) and low (−1 *SD*) number of books in the childhood home; however, individuals with more access to childhood books lived a greater proportion of this time without cognitive impairment.

**Discussion and Implications:**

Findings suggest that access to cognitive resources in childhood is protective for cognitive aging processes in older adulthood.

**Translational Significance:** With no known cure for dementia, identifying factors that may help delay the onset of cognitive impairment is imperative. Although lifestyle factors such as education appear to support cognitive resilience, the influence of childhood circumstances is less clear. Our findings indicate that older adults who had higher access to childhood books were less likely to develop cognitive impairment and had later onset relative to those with fewer available reading materials. Greater access to books in childhood represents a modifiable target for increasing resilience. Social and community programs supporting access to books may yield enduring benefits for cognitive health across the life span.

Based on population aging trends, it has been estimated that the number of people living with dementia will triple by 2050, eventually surpassing 150 million cases worldwide (Alzheimer’s Association, 2022; Nichols et al., 2022). Although there is not yet a cure for Alzheimer’s disease (AD) or related dementias, there is extensive heterogeneity in cognitive performance across middle-to-older adulthood, such that some individuals decline quickly, whereas others are able to maintain cognitive performance late into older adulthood (Mungas et al., 2010). These individual differences in cognitive functioning suggest that some individuals are more resilient in the face of cognitive aging and neuropathology characteristics of dementia (Stern et al., 2019). A number of distinct but related processes have been proposed to underlie the phenomenon of cognitive resilience (e.g., brain reserve, cognitive reserve, compensation, and brain maintenance; Stern et al., 2020).

Importantly, both observational and interventional studies suggest that these individual differences in cognitive resilience are, to some extent, malleable and bolstered by engagement in social, physical, and cognitively stimulating activities (Fratiglioni et al., 2004). For instance, physically and cognitively stimulating activities are associated with a decreased risk of dementia onset (Hersi et al., 2017). Furthermore, leisure activities in midlife (Gow et al., 2017) and older adulthood (Petkus & Gomez, 2021) are positively associated with cognitive functioning in older adulthood, whereas social activity in late life is associated with a decreased risk of cognitive impairment (Glei et al., 2005) and cognitive decline (James et al., 2011). Several similar theories propose that stimulation across the life span contributes to the maintenance and optimization of cognitive functioning in older adulthood, including cognitive enrichment, cognitive maintenance, and the “use it or lose it” hypotheses (Hertzog, 2009; Hertzog et al., 2008; Stern et al., 2019).

Cognitive ability at 8 years old was the strongest correlate of cognitive performance more than four decades later (Richards & Sacker, 2003). Likewise, research suggests that dementia risk at age 75+ years was significantly elevated for individuals with low school performance at age 9–10, adjusting for subsequent education and occupational complexity. More recently, research based on data from the Survey of Health, Ageing, and Retirement in Europe (SHARE) examined the impact of childhood socioeconomic status (SES) on trajectories of cognitive functioning in older adults aged 50 years and older (Dekhtyar et al., 2015). Findings suggested that individuals with the highest childhood SES, assessed using the retrospective lifestyle questionnaires, had better cognitive functioning in older adulthood, as well as faster rates of cognitive decline, consistent with the cognitive reserve hypothesis. However, the majority of work examining protective factors for cognitive impairment and decline later in life does not account for death as a competing risk factor, which is critical given that cognitive decline increases risk of death (Aartsen et al., 2019; Rajan et al., 2014), and that risk of both cognitive impairment and death increase alongside older age.

Overall, this work highlights the importance of childhood factors for cognitive outcomes across the life span, yet further research is needed to clarify the extent to which individual differences in childhood experiences may impact cognitive resilience in older adulthood. Further, the association between cognitive resilience and cognitive activity has been investigated by measuring the frequency of reading in middle-to-older adulthood (Casaletto et al., 2020; Lachman et al., 2010; Sattler et al., 2012; Wilson et al., 2007, 2010). In contrast, relatively few studies have examined the importance of access to cognitive resources in childhood for cognitive resilience. That is, although previous findings highlight the importance of childhood cognitive ability, school performance, and childhood SES, the extent to which access to cognitively stimulating resources contributes to cognitive resilience later in life is less clear. As two exceptions, recent work examining the impact of early-life SES, cognitive resources, and cognitive engagement on AD pathology found that more cognitive resources were associated with lower global AD pathology, as well as less cognitive decline (Oveisgharan et al., 2020), providing evidence for the brain maintenance model. The authors posited that greater access to cognitive resources in childhood (e.g., availability of materials such as books, encyclopedias, or globes in the home) represents a proxy for early-life cognitive enrichment, which may lead to downstream benefits for cognitive resilience. Further, Wilson et al. (2005) found that past cognitive activity (assessed in childhood, young adulthood, and middle age) was positively associated with cognitive functioning in older adulthood, though the association was mitigated for some cognitive domains after adjusting for current cognitive activity. However, these studies were based on relatively small samples (*N* = 813 and *N* = 576, respectively) or did not examine the independent contributions of childhood SES and access to cognitive resources.

Overall, this work highlights the importance of childhood factors for cognitive outcomes across the life span, yet further research is needed to clarify the extent to which individual differences in childhood experiences may impact cognitive resilience in older adulthood. Further, the association between cognitive resilience and cognitive activity has been investigated by measuring the frequency of reading in middle-to-older adulthood (Casaletto et al., 2020; Lachman et al., 2010; Sattler et al., 2012; Wilson et al., 2007, 2010). In contrast, relatively few studies have examined the importance of access to cognitive resources in childhood for cognitive resilience. That is, although previous findings highlight the importance of childhood cognitive ability, school performance, and childhood SES, the extent to which access to cognitively stimulating resources contributes to cognitive resilience later in life is less clear. As two exceptions, recent work examining the impact of early-life SES, cognitive resources, and cognitive engagement on AD pathology found that more cognitive resources and engagement were associated with lower global AD pathology, as well as less cognitive decline (Oveisgharan et al., 2020), providing evidence for the brain maintenance model. Further, Wilson et al. (2005) found that past cognitive activity (assessed in childhood, young adulthood, and middle age) was positively associated with cognitive functioning in older adulthood, though the association was mitigated for some cognitive domains after adjusting for current cognitive activity. However, these studies were based on relatively small samples (*N* = 813 and *N* = 576, respectively) or did not examine the independent contributions of childhood SES and access to cognitive resources.

Given the importance of understanding protective factors for cognitive aging processes in older adulthood, the current work investigates the importance of cognitive resources in early life on transitions through cognitive status categories (no cognitive impairment and impaired cognitive functioning), and death. At a clinical level, evidence for cognitive resilience may assist in providing justification and motivation for individuals to engage in stimulating activities across the entire life span. Importantly, delaying the onset of dementia by as little as 1 year is projected to reduce the number of dementia cases in 2050 by over 9 million, and would substantially decrease the global financial burden associated with dementia care (Brookmeyer et al., 2007; Zissimopoulos et al., 2015). Based on previous research and consistent with cognitive resilience theories, we hypothesized that access to more cognitive resources in childhood would be associated with a decreased risk of transitioning from no impairment to cognitive impairment, as well as more years of cognitive health span.

## Method

### Participants

Data were drawn from the SHARE, a longitudinal panel study of European adults over age 50. The SHARE project was initiated in 2004, and includes population-representative cohorts of middle and older adults throughout Europe assessed every 2 years on a variety of economic, health, and cognitive outcomes. As of the most recent eighth wave in 2020, the SHARE project has expanded to include data from 29 countries. Additional details on the sample and methodology are provided in depth elsewhere (Börsch-Supan et al., 2013). SHARE was approved by the Ethics Committee of the University of Mannheim and the Max Planck Society. More information on ethical procedures can be found on the SHARE website (https://share-eric.eu/data/data-documentation).

The present project includes data from 11 countries with at least six waves of data collection as of the most recent wave, which represents follow-up periods between 12 and 16 years. Participants were eligible for the current study if they were age 60 years or older at baseline, had at least two measurement occasions, and had available demographic and retrospective life-history information. The resulting sample included 32,783 participants from 11 countries: Austria, Belgium, Czechia, Denmark, France, Germany, Italy, the Netherlands, Spain, Sweden, and Switzerland. Demographic characteristics for the individual countries and pooled overall sample are displayed in Table 1.

**Table 1.** Baseline Demographic Characteristics

Country	*N*	*N* deaths	Sex	Age	Age at death	Cognition	Education	Childhood SES
				Mean (*SD*) [Range]	Mean (*SD*) [Range]	Mean (*SD*) [Range]	Mean (*SD*) [Range]	Mean (*SD*) [Range]
Austria	2,996	269	57.34%	70.03 (7.15) [60–93]	82.43 (8.18) [62–103]	15.32 (4.60) [0–31.80]	9.14 (4.53) [0–35]	−0.12 (0.98) [−1.70 to 4.69]
Belgium	3,429	435	54.33%	70.81 (7.59) [60–97]	83.86 (7.72) [61–103]	13.78 (4.08) [0–26.10]	11.67 (3.87) [0–25]	0.41 (1.11) [−1.70 to 9.08]
Czechia	3,788	531	57.34%	69.59 (6.94) [60–100]	79.83 (8.25) [62–100]	14.28 (3.99) [0–27.40]	12.00 (3.14) [0–25]	−0.11 (0.84) [−1.70 to 3.96]
Denmark	2,146	387	52.70%	70.13 (7.41) [60–99]	83.06 (8.13) [62–102]	15.26 (3.97) [0–27.20]	12.43 (3.74) [0–25]	0.42 (1.06) [−1.48 to 6.32]
France	2,957	304	56.88%	70.97 (7.74) [60–95]	83.88 (7.88) [62–103]	13.18 (4.08) [0–25.60]	10.66 (3.84) [0–25]	−0.19 (1.03) [−1.70 to 6.19]
Germany	2,979	214	48.64%	69.68 (6.94) [60–100]	79.97 (8.13) [61–102]	14.82 (3.92) [0–26.60]	12.26 (3.50) [0–25]	0.10 (0.93) [−1.70 to 5.87]
Italy	3,321	476	51.19%	69.49 (6.84) [60–99]	82.33 (7.53) [63–101]	12.10 (4.03) [0–28.10]	7.59 (4.32) [0–25]	−0.23 (0.90) [−1.70 to 5.99]
Netherlands	1,813	74	50.91%	68.83 (6.67) [60–90]	80.19 (8.07) [65–95]	14.88 (3.79) [0–27.10]	10.85 (3.60) [0–25]	0.35 (0.98) [−1.70 to 10.33]
Spain	4,247	810	53.14%	72.03 (8.07) [60–100]	84.64 (7.70) [63–107]	10.74 (4.21) [0–23.30]	7.63 (4.98) [0–30]	−0.25 (0.78) [−1.70 to 7.37]
Sweden	3,119	341	51.20%	70.15 (7.22) [60–95]	84.15 (8.11) [64–103]	15.14 (3.83) [0–27.20]	11.00 (4.01) [0–25]	0.52 (1.03) [−1.70 to 4.76]
Switzerland	1,988	182	51.91%	70.27 (7.25) [60–95]	83.34 (7.60) [64–101]	15.23 (3.76) [1.80–26.80]	8.41 (5.10) [0–25]	0.76 (1.00) [−1.70 to 8.48]
Overall sample	32,783	4,023	53.45%	70.28 (7.36) [60–100]	82.86 (8.07) [61–107]	13.62 (4.40) [0–31.80]	10.28 (4.47) [0–35]	0.00 (1.00) [−1.70 to 10.33]

*Notes*: *N* = sample size; *N* Deaths reflect number of total deaths across all follow-up occasions; Sex reflects percentage of female participants. Socioeconomic status (SES), Age at death, and Education are reported in years. *SD* = standard deviation.

### Measures

#### Access to books at age 10

Participants completed retrospective life-history reports in the third wave of SHARE data collection. As part of this life-history report, participants were asked to indicate the approximate number of books within their household at age 10 years, excluding magazines, newspapers, and school books. Participants reported the number of books on a five-point scale; 1 = none or very few (~0–10 books), 2 = enough to fill one shelf (~11–25 books), 3 = enough to fill one bookcase (26–100 books), 4 = enough to fill two bookcases (101–200 books), and 5 = enough to fill two or more bookcases (>200). Supplementary Table 1 presents responses to this measure across countries. This question was repeated approximately 4 years later in a subsample of 8,405 participants as part of the Wave 5 assessment. Reports from these two measurement occasions were very highly correlated (*r* = 0.998, *p* < .001), suggesting good reliability of these retrospective estimates. Numerical values were assigned to each category, corresponding to the lower bound of the numerical book estimate (i.e., 1 = 0 books, 2 = 11 books, etc.). Mean values were used in cases where participants had discrepant estimates across measurement occasions. Book estimates were then standardized within each country using *z*-transformation to reflect each participant’s relative position to their own country’s mean in standard deviation (*SD*) units.

#### Assessment of cognition and death status

Participants were classified at each wave as having normal cognitive functioning or cognitive impairment based on scores from a modified version of the Telephone Interview of Cognitive Status (TICS); a brief nondiagnostic assessment comparable to the Mini-Mental State Examination, and in this case, administered in person (Brandt et al., 1988). Participants were administered word recall tasks involving recall of a 10-word list immediately and after a delay, as well as tests of date orientation (year, month, day, day of week) and a verbal fluency task consisting of naming as many animals correctly as possible during a 60-s period. Scores on the animal naming fluency task were standardized to a 0–10 scale to align with the word recall tasks. Scores from these tasks were summed for each individual (possible range: 0–34) and used to compute country-specific *z*-scores at the baseline wave. Individuals were classified as having cognitive impairment if they scored 1.5 *SD*s or more below their country-level mean, and normal cognitive functioning if they scored above this value. To provide consistent scaling across time and to account for potential mean-level changes in cognitive functioning over the follow-up period, cutoff values for the classification of cognitive impairment from the baseline wave were used for all subsequent assessments.

SHARE requests that the interviewers confirm the death of a respondent by a proxy respondent. In case of death, interviewers conducted an end-of-life interview with a proxy respondent, who could be a family member, a household member, a neighbor, or any other person in the closer social network of the deceased respondent. The end-of-life interview mainly involved information on life circumstances in the year before the respondent died, as well as the circumstances of death (e.g., time/cause of death).

#### Covariates

Age, sex, education, and childhood SES were included as covariates. Age was centered at the baseline overall sample mean of 74 years to facilitate comparisons of effect sizes across countries. Education was measured in years and centered on the country-specific mean. Childhood SES was assessed via the number of rooms in the house (excluding kitchen, bathrooms, and hallways), number of facilities in the house (fixed bath, cold running water supply, hot running water supply, inside toilet, and central heating), and occupation of the main household breadwinner at age 10. Occupation of the main breadwinner was categorized based on International Standard Classification of Occupation (International Labour Office, 2012) skill levels: elementary occupations (laborers, cleaners, food preparation workers), skilled occupations (service or sales workers, skilled agricultural or fishery workers, trades workers, machine operators, or assembly workers), associate occupations (technician or associate professionals, clerical or support workers), and managerial occupations (managers, senior officials, or legislators, other professionals). Values were assigned to each classification ranging from 0 (elementary occupations) to 3 (managerial occupations). Consistent with previous research examining childhood conditions in the SHARE sample (Mazzonna, 2014), principal components analysis (PCA) was used to reduce the dimensions of the multiple SES indicators. A single component from the PCA based on the correlation matrix was found to exceed an eigenvalue greater than 1 (eigenvalue = 1.56, explained variance = 0.52) and was retained as a single index of childhood SES. Two other components had low eigenvalues of 0.79 and 0.64 and were not retained. Component loadings for the single SES index were 0.67 for parental occupation, 0.71 for number of rooms, and 0.78 for facilities.

#### Statistical analysis

**Figure 1.**
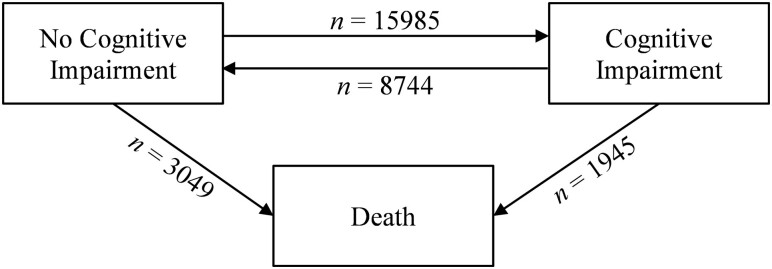
Three-state model for cognitive functioning and death. Values indicate the number of observed transitions (*n*) between states in the overall sample across up to 16 years follow-up.

Multistate survival models (MSMs) were used to examine transitions across cognitive status categories and death over time using the MSM package in R (Jackson, 2011). Three-state models were specified, which provides the opportunity to simultaneously model transitions between two cognitive status categories (normal cognitive functioning and cognitive impairment), while controlling for death as a competing risk (Figure 1). Further, SHARE is a longitudinal panel study with fixed biennial assessment; thus, although cognitive declines occur in continuous time, prescheduled surveys restrict measurement of the timing of cognitive changes. MSM is favorable when data are interval censored because there are no assumptions about time spent in each state.

To complement the MSM analyses, we estimated years without cognitive impairment (i.e., cognitive health span) and total life expectancies (LEs), conditional on age, using the ELECT package (van den Hout et al., 2019). The package fits a multinomial regression model using the transition probabilities estimated by the MSM for time-invariant covariates. Thus, LEs for the total sample were estimated for male and female participants at 65, 70, 75, and 80 years of age. For these estimates, covariates were held constant at country-specific mean SES value, as well as mean age and education (in years). However, cognitive health span and total longevity were estimated for individuals who had high (+1 *SD*) and low (−1 *SD*) access to books in the home at age 10 years to provide an estimate of the effect of access to books in childhood on mortality.

## Results

### Risk for Cognitive Impairment and Death

#### Pooled analyses

**Figure 2.**
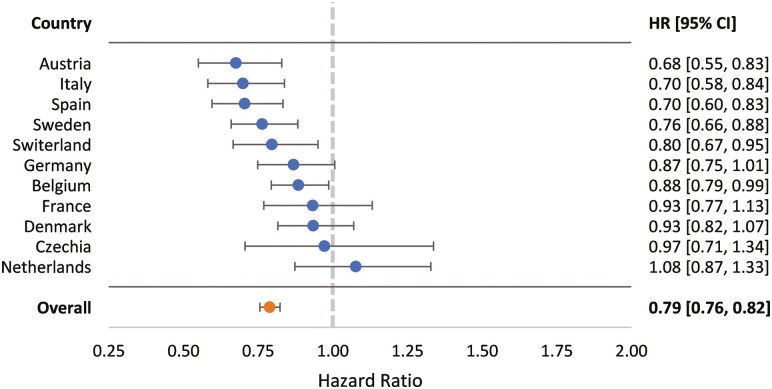
Adjusted associations of the number of books in the home at age 10 with late-life cognitive impairment risk. Models adjusted for baseline age, sex, years of education, and childhood socioeconomic standing. HR = hazard ratio for each 1 standard deviation change in childhood books; 95% CI = 95% confidence intervals for each estimate.

In the pooled overall sample, having access to more books at age 10 was associated with a decreased risk of developing cognitive impairment after adjusting for age, sex, education, and childhood SES. Each *SD* increase in reported books in the home predicted approximately a 21% decreased risk of cognitive impairment (adjusted hazard ratio [HR_adj_] = 0.79, confidence interval [CI]: 0.76–0.82). Figure 2 displays HRs for the transition from normal cognition to cognitive impairment for the overall sample, as well as country-specific estimates. In addition to books, synthesized estimates for the overall sample suggested that female participants and those higher in education were at a reduced risk of developing cognitive impairment, whereas participants who were older or had higher childhood SES were at increased risk (Table 2).

**Table 2.** Hazard Ratios for the Effect of Variables on Transitions of Older Adults Through Cognitive Functioning Categories and Death

Transition	NCI—CI	NCI—Death	CI—NCI	CI—Death
Age, years	1.04 (1.04, 1.05)	1.13 (0.91, 1.40)	0.96 (0.95, 0.96)	1.07 (1.06, 1.08)
Sex, female	0.84 (0.79, 0.89)	0.36 (0.09, 1.36)	1.01 (0.93, 1.09)	0.77 (0.67, 0.89)
Education, years	0.96 (0.96, 0.97)	0.96 (0.76, 1.20)	1.02 (1.01, 1.02)	1.02 (1.00, 1.03)
Childhood SES	1.06 (1.02, 1.10)	1.31 (0.93, 1.86)	1.13 (1.08, 1.18)	0.83 (0.78, 0.88)
Books at age 10	0.79 (0.75, 0.83)	1.58 (0.94, 2.65)	0.95 (0.90, 1.00)	1.10 (0.97, 1.23)

*Notes*: NCI = no cognitive impairment; CI = cognitive impairment; SES = socioeconomic status. Values indicate hazard ratios with 95% confidence intervals in parentheses.

#### Country-specific results

Effect sizes ranged from 0.68 in Austria (CI: 0.55–0.83) to 1.08 in the Netherlands (CI: 0.97–1.33). Ten of the 11 countries had HR estimates that were below 1.00, with 6 of these effects having CIs that did not cross the 1.00 threshold, suggesting a protective effect of childhood books on risk for cognitive impairment. Conversely, access to books in the home at age 10 did not predict mortality risk for the transition from normal cognitive functioning to death or for the transition from cognitive impairment to death.

### Life Expectancies

**Figure 3.**
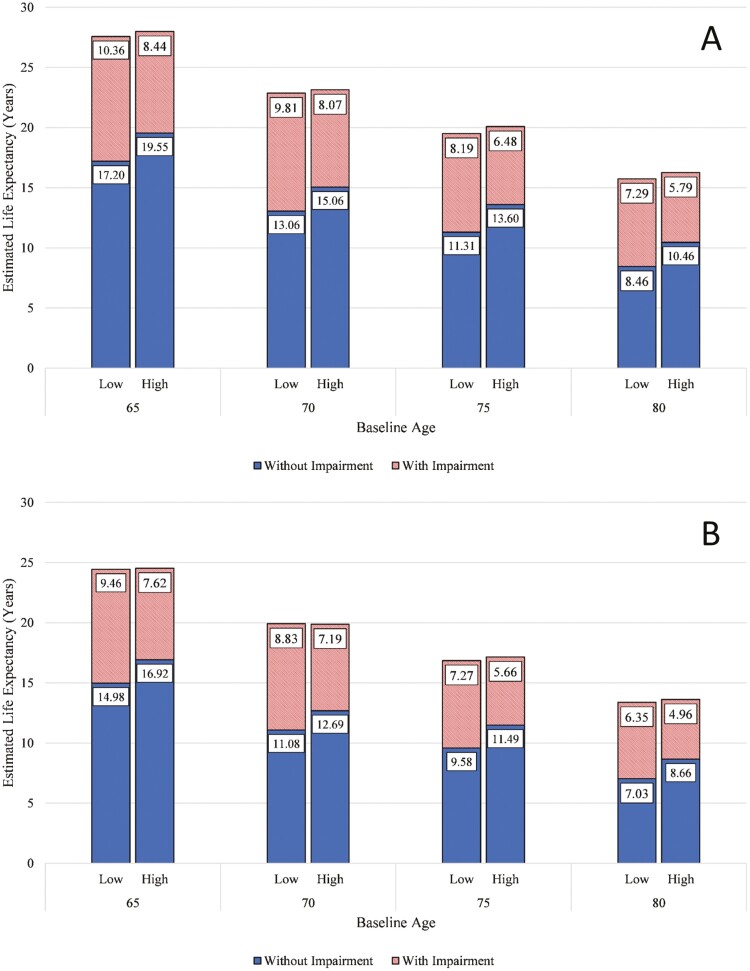
Estimated life expectancies for (A) female participants and (B) male participants comparing high (+1 standard deviation) and low (−1 standard deviation) number of books in the home at age 10. Dashed bars represent the portion of remaining life expectancy with cognitive impairment.

Results from the multistate models were used to compute LEs for ages 65, 70, 75, and 80 at baseline, with overall LEs divided between normal cognitive function and cognitive impairment. LEs for female and male participants are displayed in Figure 3. Overall LEs were similar between participants reporting high (+1 *SD*) and low (−1 *SD*) number of books in the childhood home; however, individuals with higher access to childhood books on average lived a greater proportion of this time without cognitive impairment. For instance, among women aged 80 at baseline, those with higher numbers of books in the childhood home lived on average 2 years longer free of cognitive impairment relative to individuals lower in childhood books despite similar overall LEs of around 16 years. LE estimates for men were consistent with this pattern, but with lower overall LEs.

## Discussion

Using a large representative sample of older adults from 11 European countries, this project investigated whether access to cognitively stimulating materials in the childhood home is differentially associated with cognitive impairment, cognitive health +span, and mortality in older adulthood. Results from the overall sample revealed that participants who reported having more books at age 10 had a decreased likelihood of transitioning from no impairment to cognitive impairment over up to 16 years after adjustment for childhood SES and formal education. Further, individuals with access to more books in childhood were expected to live longer without cognitive impairment—over 2 years longer in participants aged 65 at baseline—relative to those with less access to books, despite no differences in total LEs. These findings suggest that environmental enrichment in childhood may support cognitive resilience, resulting in the extension of cognitive health span and compression of morbidity in those who eventually develop cognitive impairment. Our findings highlight the need for future research efforts focused on uncovering the mechanisms underlying these associations. For instance, access to books in childhood may initiate an investment in learning during a critical period of brain development. Similarly, access to cognitively stimulating materials in childhood may support exposure to various ideas and stories, encouraging a variety of interests and a sense of curiosity.

In recent years, there have been increased calls to investigate potentially modifiable contributors to cognitive resilience beyond established indicators such as education or occupational complexity (Schwartz et al., 2016; Stern et al., 2019). Indeed, some have suggested that an over-reliance on education in early adulthood as a singular proxy for resilience processes may fail to capture critical periods in the development of cognitive resilience capacity (Chapko et al., 2018). Directly addressing this call, this project focuses on a modifiable factor (i.e., access to reading materials) while taking years of education into account. Furthermore, although recent work has identified education as a prominent modifiable risk factor for dementia in low- and middle-income countries (Mukadam et al., 2019), the resources required to increase access to education pose a substantial barrier. Conversely, amplifying access to cognitively stimulating materials in childhood represents an exciting opportunity to promote cognitive resilience across the entire lifespan through policy or community efforts. Importantly, our results suggest that access to books is not only important for cognitive functioning in older adulthood when adjusting for education, but also may offer comparatively more protection. That is, when considering the transition from no cognitive impairment to cognitive impairment, synthesized results suggest that 1 *SD* higher in education is associated with approximately 14% reduced risk (HR = 0.86, 95% CIs = 0.83, 0.89), whereas 1 *SD* higher in access to books is associated with approximately 21% reduced risk (HR = 0.79, 95% CIs = 0.75, 0.83). Moreover, these analyses adjust for various indicators of childhood household affluence, including number of rooms and facilities in the home and parental occupation.

A primary strength of our analytic approach is the ability to evaluate both generalizability and variability in results across countries. As shown in Figure 2, the effect of access to books in childhood on the transition from no impairment to cognitive impairment is fairly consistent across countries, with a few minor exceptions. Namely, although the effect size for the Netherlands is not significantly different from zero, the direction of the effect is in the opposite direction as the other countries. This may be due to limited individual transitions between states and/or only 4% mortality across follow-up occasions (see Supplementary Table 2 and Table 1, respectively). Imprecision in estimates may also be due to culture-specific constructs that are unaccounted for in the model (e.g., famine or malnutrition sustained during the Dutch Famine, which occurred when these participants were 9–10 years old, on average; Halmdienst & Winter-Ebmer, 2014). Further, although Czechia was the largest sample, the effect is characterized by extensive CIs, which may be due to availability of only six (rather than seven) measurement occasions.

The current study is characterized by numerous strengths, including a large sample of over 32,000 participants followed for up to 16 years across 11 European countries and adjustment for established determinants of adult cognitive functioning and childhood SES while considering mortality as a competing risk factor; however, some limitations should be considered when interpreting our findings and informing future-related investigations. First, although repeated assessments of the number of books in childhood were highly consistent within participants and effort was made to adjust for discrepant reporting, retrospective accuracy over such a long period is unclear. Longitudinal evaluation of retrospective reports suggests that estimates of physical or environmental characteristics (e.g., number of books or size of book shelving) are more accurate than retrospective reports of psychosocial factors, but may, nevertheless, introduce retrospective bias when follow-up occurs over several years (Henry et al., 1994). Second, SHARE did not collect information on the extent to which participants were interacting with these reading materials. Thus, although the present study discussed access to books in the childhood home as an indicator of cognitive enrichment, it is possible that alternative pathways may account for the observed associations with cognitive health in older adulthood. Future research in this area would benefit from examining the degree of engagement to better understand the implications of access to cognitive resources on neurocognitive development.

Third, although TICS has been used extensively to assess cognitive functioning (Yoneda et al., 2021), the modified version used in the present sample does not have established clinical cutoffs and may be less sensitive for detecting cognitive impairment relative to more established measures or a full neuropsychological battery. Fourth, although the current project includes data representing diverse participants across 11 European countries, generalizability is limited to the represented generations (e.g., children born in the 1930s, on average) and race. Emerging research suggests that dementia and cognitive decline unfold differently for Black and Mexican populations (Mehta & Yeo, 2017); as such, researchers should make concerted efforts to collect longitudinal cognitive and psychosocial data on historically marginalized groups. Finally, we only investigated a three-state model given limited variability and floor effects in cognitive functioning scores in some countries. Future research should examine how access to books in childhood is related to more nuanced metrics of change in cognitive functioning.

Together, our findings suggest that access to books in childhood may produce enduring benefits for cognitive health in older adulthood (i.e., enhanced cognitive health span), which may thereby optimize well-being and independence in older adulthood. Furthermore, our findings emphasize the value of a lifespan approach to cognitive resilience. Our findings are consistent with theory (e.g., the cognitive reserve hypothesis) and experimental research focused on the influence of enriched learning environments (Mate et al., 2022; Rose et al., 2019) supporting both ecological validity and the global need to identify modifiable lifestyle factors that contribute to cognitive resilience in older adulthood.

## Supplementary Material

igad124_suppl_Supplementary_Tables_S1-S2

## Data Availability

SHARE data has been made publicly available and information on data access can be found online (http://www.share-project.org/data-access.html). This project was not preregistered, but analytic script has been made available at https://osf.io/wzgj4/.
