# Supplementary material for: Availability of Cognitive Resources in Early Life Predicts Transitions Between Cognitive States in Middle and Older Adults From Europe
Source: Innov Aging. 2023 Oct 26;7(9):igad124. doi: 10.1093/geroni/igad124 (PMC10682867; doi:10.1093/geroni/igad124)
Supplement: igad124_suppl_Supplementary_Tables_S1-S2 [file igad124_suppl_supplementary_tables_s1-s2.docx]

*Innovation in Aging* Online Supplementary Material: Nathan A. Lewis, Tomiko Yoneda, René J. F. Melis, Daniel K. Mroczek, Scott M. Hofer, & Graciela Muniz-Terrera. Availability of cognitive resources in early life predicts transitions between cognitive states in middle and older adults from Europe.

Supplemental Table 1. Access to books in childhood at age 11 years

| Country | 0-10 Books | 11-25 Books | 26-100 Books | 101-200 Books | 201> Books |
| --- | --- | --- | --- | --- | --- |
|  | *N* (%) | *N* (%) | *N* (%) | *N* (%) | *N* (%) |
| Austria | 1101 (36.75%) | 831 (27.74%) | 577 (19.26%) | 271 (9.05%) | 216 (7.21%) |
| Belgium | 1486 (43.34%) | 777 (22.67%) | 657 (19.16%) | 281 (8.19%) | 228 (6.65%) |
| Czechia | 561 (14.81%) | 1013 (26.74%) | 1280 (33.79%) | 531 (14.02%) | 403 (10.64%) |
| Denmark | 484 (22.55%) | 496 (23.11%) | 629 (29.31%) | 267 (12.44%) | 270 (12.58%) |
| France | 1518 (51.34%) | 680 (23.00%) | 459 (15.52%) | 172 (5.82%) | 128 (4.33%) |
| Germany | 954 (32.02%) | 888 (29.81%) | 689 (23.13%) | 258 (8.66%) | 190 (6.38%) |
| Italy | 2273 (68.44%) | 616 (18.55%) | 310 (9.33%) | 79 (2.38%) | 43 (1.29%) |
| Netherlands | 654 (36.07%) | 474 (26.15%) | 429 (23.66%) | 125 (6.90%) | 131 (7.23%) |
| Spain | 2359 (55.55%) | 1213 (28.56%) | 464 (10.93%) | 130 (3.06%) | 81 (1.91%) |
| Sweden | 597 (19.14%) | 751 (24.08%) | 1028 (32.96%) | 398 (12.76%) | 345 (11.06%) |
| Switzerland | 573 (28.82%) | 562 (28.27%) | 493 (24.80%) | 193 (9.71%) | 167 (8.40%) |
| *Overall* | 12560 (38.31%) | 8301 (25.32%) | 7015 (21.40%) | 2705 (8.25%) | 2202 (6.72%) |

Supplemental Table 2. Individual transitions between states

| Country | State 1 to 2 | State 1 to 3 | State 2 to 1 | State 2 to 3 |
| --- | --- | --- | --- | --- |
|  | *n* | *n* | *n* | *n* |
| Austria | 1131 | 155 | 575 | 114 |
| Belgium | 1237 | 244 | 593 | 191 |
| Czechia | 1693 | 319 | 968 | 212 |
| Denmark | 934 | 209 | 487 | 178 |
| France | 1184 | 170 | 637 | 134 |
| Germany | 1016 | 141 | 580 | 72 |
| Italy | 1284 | 289 | 678 | 184 |
| Netherlands | 524 | 51 | 206 | 23 |
| Spain | 1348 | 526 | 678 | 284 |
| Sweden | 1466 | 195 | 847 | 144 |
| Switzerland | 976 | 100 | 620 | 82 |
| Overall | 12793 | 2399 | 6869 | 1618 |

*Note*. *n* = number of individual transitions between states; State 1 = No cognitive impairment; State 2 = Cognitive impairment; State 3 = Death
